# Global epidemiology of avian influenza A(H5N1) virus infection in humans, 1997 – 2015: a systematic review

**DOI:** 10.1016/S1473-3099(16)00153-5

**Published:** 2016-05-17

**Authors:** Shengjie Lai, Ying Qin, Benjamin J. Cowling, Xiang Ren, Nicola A. Wardrop, Marius Gilbert, Tim K. Tsang, Peng Wu, Luzhao Feng, Hui Jiang, Zhibin Peng, Jiandong Zheng, Qiaohong Liao, Sa Li, Peter W. Horby, Jeremy J. Farrar, George F. Gao, Andrew J. Tatem, Hongjie Yu

**Affiliations:** Division of Infectious Disease, Key Laboratory of Surveillance and Early–warning on Infectious Disease, Chinese Center for Disease Control and Prevention, Beijing, China; Department of Geography and Environment, University of Southampton, Southampton, UK; Flowminder Foundation, Stockholm, Sweden; Division of Infectious Disease, Key Laboratory of Surveillance and Early–warning on Infectious Disease, Chinese Center for Disease Control and Prevention, Beijing, China; WHO Collaborating Centre for Infectious Disease Epidemiology and Control, School of Public Health, Li Ka Shing Faculty of Medicine, The University of Hong Kong, Hong Kong Special Administrative Region, China; Division of Infectious Disease, Key Laboratory of Surveillance and Early–warning on Infectious Disease, Chinese Center for Disease Control and Prevention, Beijing, China; Department of Geography and Environment, University of Southampton, Southampton, UK; Biological Control and Spatial Ecology, Universite′ Libre de Bruxelles, Brussels, Belgium; Fonds National de la Recherche Scientifique, Brussels, Belgium; WHO Collaborating Centre for Infectious Disease Epidemiology and Control, School of Public Health, Li Ka Shing Faculty of Medicine, The University of Hong Kong, Hong Kong Special Administrative Region, China; WHO Collaborating Centre for Infectious Disease Epidemiology and Control, School of Public Health, Li Ka Shing Faculty of Medicine, The University of Hong Kong, Hong Kong Special Administrative Region, China; Division of Infectious Disease, Key Laboratory of Surveillance and Early–warning on Infectious Disease, Chinese Center for Disease Control and Prevention, Beijing, China; Division of Infectious Disease, Key Laboratory of Surveillance and Early–warning on Infectious Disease, Chinese Center for Disease Control and Prevention, Beijing, China; Division of Infectious Disease, Key Laboratory of Surveillance and Early–warning on Infectious Disease, Chinese Center for Disease Control and Prevention, Beijing, China; Division of Infectious Disease, Key Laboratory of Surveillance and Early–warning on Infectious Disease, Chinese Center for Disease Control and Prevention, Beijing, China; Division of Infectious Disease, Key Laboratory of Surveillance and Early–warning on Infectious Disease, Chinese Center for Disease Control and Prevention, Beijing, China; Division of Infectious Disease, Key Laboratory of Surveillance and Early–warning on Infectious Disease, Chinese Center for Disease Control and Prevention, Beijing, China; Oxford University Clinical Research Unit, Wellcome Trust Major Overseas Programme, Ho Chi Minh City, Vietnam; Centre for Tropical Medicine, Nuffield Department of Clinical Medicine, Oxford University, UK; Singapore Infectious Disease Initiative, Singapore; Oxford University Clinical Research Unit, Wellcome Trust Major Overseas Programme, Ho Chi Minh City, Vietnam; Centre for Tropical Medicine, Nuffield Department of Clinical Medicine, Oxford University, UK; Singapore Infectious Disease Initiative, Singapore; ISARIC, Centre for Tropical Medicine, University of Oxford, Churchill Hospital, United Kingdom; CAS Key Laboratory of Pathogenic Microbiology and Immunology, Institute of Microbiology, Chinese Academy of Sciences, Beijing, China; Office of Director-General, Chinese Center for Disease Control and Prevention, Beijing, China; Department of Geography and Environment, University of Southampton, Southampton, UK; Flowminder Foundation, Stockholm, Sweden; Fogarty International Center, National Institutes of Health, Bethesda, MD, USA; Division of Infectious Disease, Key Laboratory of Surveillance and Early–warning on Infectious Disease, Chinese Center for Disease Control and Prevention, Beijing, China

## Abstract

Avian influenza viruses A(H5N1) have caused a large number of typically severe human infections since the first human case was reported in 1997. However, there is a lack of comprehensive epidemiological analysis of global human cases of H5N1 from 1997-2015. Moreover, few studies have examined in detail the changing epidemiology of human H5N1 cases in Egypt, especially given the most recent outbreaks since November 2014 which have the highest number of cases ever reported globally over a similar period. Data on individual cases were collated from different sources using a systematic approach to describe the global epidemiology of 907 human H5N1 cases between May 1997 and April 2015. The number of affected countries rose between 2003 and 2008, with expansion from East and Southeast Asia, then to West Asia and Africa. Most cases (67.2%) occurred from December to March, and the overall case fatality risk was 53.5% (483/903) which varied across geographical regions. Although the incidence in Egypt has increased dramatically since November 2014, compared to the cases beforehand there were no significant differences in the fatality risk , history of exposure to poultry, history of human case contact, and time from onset to hospitalization in the recent cases.

## INTRODUCTION

Highly pathogenic avian influenza (HPAI) A(H5N1) virus was first isolated and characterised in a domestic goose in Guangdong province, China in 1996,^1^ and outbreaks have since been reported in domestic poultry, wild birds and humans in over 60 countries.^2-4^ The spread of HPAI H5N1 in poultry populations increases the risk of human infections.^5-8^ The first reported case of human illness with H5N1 virus infection occurred in May 1997 in Hong Kong Special Administrative Region (SAR) of China, with a total of 18 cases and 6 deaths.^9-12^ After an apparent five-year absence, two cases with a history of travel to southern China were reported in February 2003 in Hong Kong SAR.^13^ Following the pattern of spread and persistence of the virus in poultry, human cases of H5N1 virus infection with high mortality were subsequently detected in China,^14,15^ Southeast Asia,^16,17^ West Asia,^18,19^ and most recently Africa, with cases detected in Egypt every year from 2006 to 2015.^20-23^ Compared to previous years,^24-26^ the incidence of human H5N1 cases has remained at a low level globally between October 2012 and October 2014,^27,28^ while attention has been focused on the more recent emergence of variant swine influenza A(H3N2) in North America,^29^ novel avian influenza A(H7N9) in China,^30-32^ other avian influenza A(H5) subtypes in Asia, Europe and North America,^27,33^ and other emerging infections.^34-36^

However, between 1 November 2014 and 30 April 2015, a total of 165 cases, including 48 deaths were reported to the World Health Organization (WHO).^37^ This is by far the highest number of human cases ever reported globally over a similar period.^38^ Moreover, the number of human H5N1 cases reported in Egypt with onset in February 2015 is the highest number reported by any country in a single month.^39^ The emergence of a novel cluster of H5N1 virus clade 2.2.1.2 has been found in poultry in Egypt since mid-2014 and has quickly become predominant.^40^ It is not yet known if this emerging phylotype has increased zoonotic potential and, thus, can be transmitted more efficiently to humans.^39-41^

There is a lack of comprehensive epidemiological analysis of global human cases of H5N1 for the 1997-2015 period,^17,42-45^ and few studies have presented in detail the changing epidemiology of human H5N1 cases in Egypt by comparing the cases before November 2014 to the most recent outbreaks from November 2014 through to April 2015.^20,40,46^ In order to improve understanding of the epidemiology of HPAI H5N1, we conducted a systematic review of individual case data to describe the magnitude and distribution of all human H5N1 cases globally with illness onset between 1 May 1997 and 30 April 2015, focusing on the characteristics of cases, seasonal and geographical patterns, and examining in more detail the epidemiology of human H5N1 cases in Egypt.

## METHODS

### Search strategy and selection criteria

Human H5N1 case data were identified and compiled according to the probable and confirmed case definitions described in the next section. Data on all human H5N1 cases in mainland China were downloaded from the online National Notifiable Infectious Disease Reporting Information System at the Chinese Center for Disease Control and Prevention (China CDC).^30,47^ Data on human H5N1 cases in Vietnam and Azerbaijan as of 30 April 2014 were provided by the Vietnam National Institute of Hygiene and Epidemiology and the Azerbaijan Ministry of Health, respectively. Data on human H5N1 cases in all other affected countries or regions were obtained from publicly available sources (Appendix Table 1), including the WHO’s Disease Outbreak News of the Global Alert and Response (GAR);^48^ the WHO’s Weekly Epidemiological Record;^49^ the WHO Western Pacific Region’s Avian Influenza Weekly Update;^50^ the FluTrackers website (www.flutrackers.com);^51^ and the websites of the Ministries of Health in individual countries or regions.

**Figure 1:**
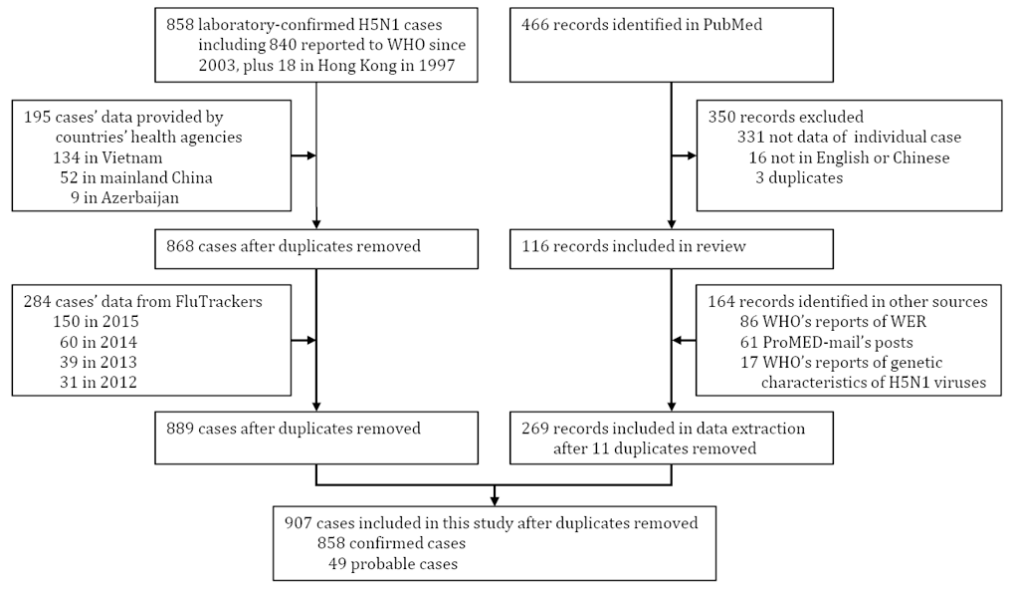
Flow chart of study selection and collection of individual case data on H5N1 cases

We also searched in PubMed for related studies using a systematic review approach that followed the PRISMA (Preferred Reporting Items for Systematic Reviews and Meta-Analyses) guidelines (Figure 1).^52^ The literature published from May 1, 1997 to April 30, 2015 was searched by the queries “(H5N1[Title] AND (PATIENT[Title] OR PATIENTS[Title] OR HUMAN[Title] OR HUMANS[Title] OR PERSON[Title] OR CASE[Title] OR CASES[Title])) AND ("1997/05/01"[Date - Publication] : "2015/04/30"[Date - Publication])”. Articles published in English and Chinese were included, and the full-text of Chinese articles was searched from China National Knowledge Infrastructure and Wanfang Data. Relevant articles and reports published between 1997 and 2015 were identified through searches in the reports from WHO and the ProMed-mail posts. Articles resulting from these searches and relevant references cited in those articles were reviewed.

WHO GAR updates and the WHO statistics on cumulative number of confirmed human H5N1 cases from November 2003 to April 2015 were used to establish a line list of human H5N1 cases.^48,53^ All cases from sources other than WHO updates were matched with the initial line list (Figure 1). The latest cases, which were not yet officially announced by WHO, were identified through ProMed-mail posts and FluTrackers, and confirmed by the announcements of Ministries of Health in individual countries/regions. When critical information was missing, additional information was sought from published literature, ProMed posts and English language news releases from the regional office of WHO and the relevant Ministry of Health (Appendix Table 1).^18,50,54-57^

### Case definition

The WHO case definition was used.^58^ A ***confirmed case*** was defined as a human case of influenza A(H5N1) virus infection reported by WHO and with laboratory confirmation, i.e. a patient with defined clinical signs, epidemiological linkage and laboratory confirmation by an influenza laboratory accepted by WHO, as specified in the WHO case definition. Other reported cases were considered as ***probable cases*** if they had exposure to other confirmed human cases, or to sick or dead poultry, or the H5N1 infection was confirmed by the country or local institutions but not meeting WHO criteria or announced by WHO.

### Data variables and extraction

All probable and confirmed cases with illness onset by 30 April 2015 were included in the analysis. Individual data on cases included age, sex, country, type of diagnosis, year, month and day of onset, date of hospitalization, final outcome (fatal or non-fatal), date of outcome, and potential risk factors (Appendix Table 2). Information on exposure potentially related to the acquisition of H5N1 infections was collected (Box 1). Where contradictory information was found for a given variable, WHO and Ministry of Health data were given priority over journal articles, and journal articles were given priority over other sources of information (Appendix Table 1). The epidemic curves were plotted and the demographic characteristics were summarized by outcome and geographical region.

Data on the clade or subclade of H5N1 virus isolated from human cases were also collated from the regular WHO reports.^59^ In total, 17 reports issued between August 2006 and February 2015 were reviewed, which provided information on H5N1 virus clades circulating and characterized from 1997 to February 2015.^59^ However, not all individual cases were reported with laboratory results of the clade or subclade, and thus, where this information was not available, the infection was presumed with the clade or subclade of H5N1 virus in the same period and area.^17,40,42,60,61^ All data used in this study were anonymized.

### Ethical approval

The National Health and Family Planning Commission of China, the Ministry of Health of Vietnam, and the Ministry of Health of Azerbaijan determined that the collection of data from human cases of avian influenza A(H5N1) virus infection was part of the public health investigation of an outbreak and was exempt from institutional review board assessment. All data were supplied and analyzed in an anonymous format, without access to personal identifying information.

### Role of the funding source

The sponsor of the study had no role in the study design, data collection, data analysis, data interpretation, writing of the report, or the decision to publish. The corresponding author had full access to all the data in the study and had final responsibility for the decision to submit for publication. The views expressed are those of the authors and do not necessarily represent the policy of the China CDC or the institutions with which the authors are affiliated.

## RESULTS

**Figure 2:**
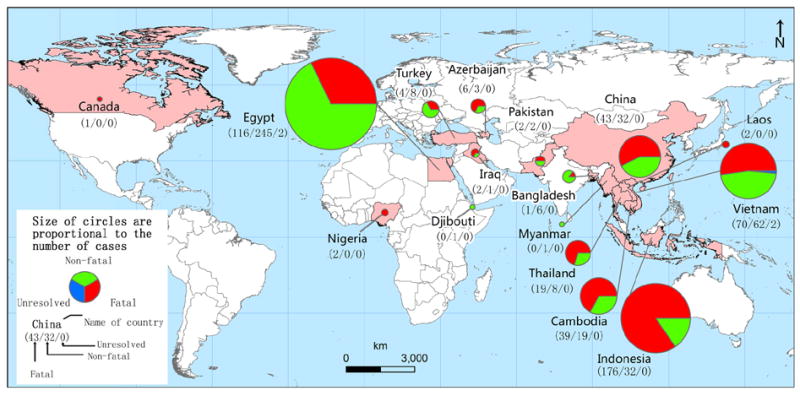
The geographic distribution of human cases with H5N1 virus infection by outcome, May 1997–April 2015 (n=907) The data for China includes the cases reported by mainland China (52 cases) and Hong Kong SAR (23 cases).

**Figure 3:**
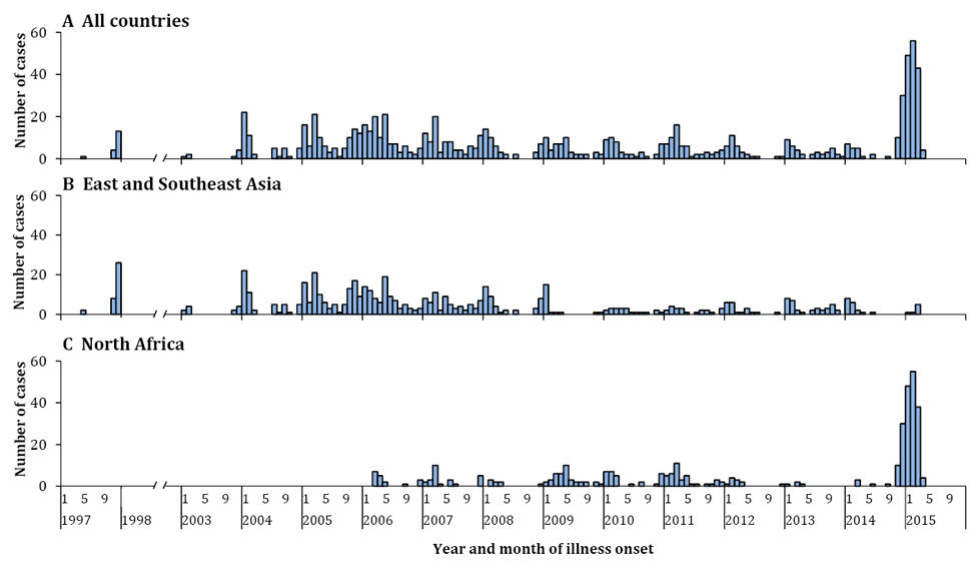
Epidemic curve of human cases with H5N1 virus infection by region, May 1997–April 2015 (A) The epidemic curve of H5N1 human cases reported globally (884 cases). (B) East and Southeast Asia (484 cases) includes Indonesia (187), Viet Nam (134), Cambodia (58), mainland China (52), Thailand (27), Hong Kong SAR (23), Laos (2), and Myanmar (1). (C) North Africa (363 cases) includes Egypt (363). Twenty-three cases with unknown month of illness (21 cases of Indonesia in 2009 and two cases of Turkey in 2006) are excluded from this epidemic curve.

A total of 907 human H5N1 cases were reported globally during the 18-year period from May 1, 1997 through April 30, 2015, of which 94.6% were confirmed cases and 5.4% were probable cases (Table 1 and Figure 2). Annual case numbers displayed striking variations, with the highest numbers recorded in 2015 (Figure 3, Appendix Figures 1-2). The total number of cases (213) in 2014-2015 was greater than that (181 cases) of the four years from 2010-2013 (Appendix Figure 3), with the highest monthly number occurring in February 2015 when there were 55 cases in Egypt and one in China.

**Figure 4:**
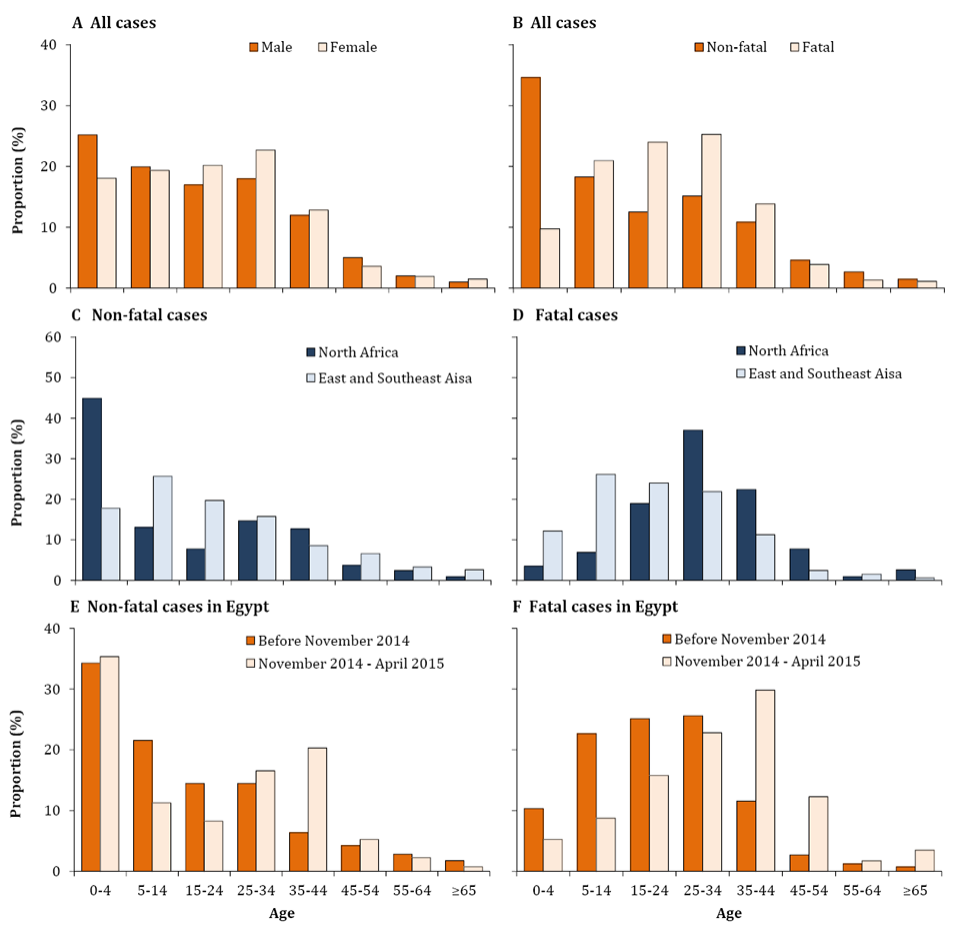
The age distribution of human cases with H5N1 virus infection by gender, geographic regions and outcome, May 1997–April 2015 (A) The age distribution of all cases by male (n=401) and female (n=476). (B) The age distribution of all cases by death (n=463) and survive (n=416). (C) The age distribution of survive cases by North Africa (n=245), East and Southeast Asia (n=152). (D) The age distribution of death cases by North Africa (n=116), East and Southeast Asia (n=329). (E) The age distribution of survive cases in Egypt before (n=114) and since 1 November 2014 (n=131). (F) The age distribution of death cases in Egypt before (n=64) and since 1 November 2014 (n=52).

The overall male-to-female ratio was almost even (1:1.2) from 1997 to 2014, although this pattern was not uniform across regions (Table 1). The median age of cases was 19 years, with an inter quartile range (IQR) from 5 to 32 years, and 41.2% (363/881) were children under 15 years of age and 80.3% (707/881) were in people under 35y. The median age of non-fatal cases was younger in North Africa and older in East and Southeast Asia (median and IQR: 6 and 3-31 years vs. 18.5 and 6-30 years), but the median age of fatal cases was older in North Africa than in East and Southeast Asia (median and IQR: 30 and 20-36 years vs. 19 and 9-30 years) (Figure 4).

In total, 16 countries reported human cases between 1997 and 2015. The number of affected countries has risen between 2003 and 2008, with expansion from East Asia to Southeast Asia, then West Asia, North Africa and other regions, and apparent ongoing transmission and cases reported almost every year in China, Vietnam, Cambodia, Indonesia and Egypt (Appendix Figure 4A). The incidence in Asia remained at low levels in 2013-2015, while the number of cases in Egypt has increased in 2014-2015. During 1997-2015, 67.2% (594/884) of cases were reported from December to March, with a peak in January (20.9%) (Appendix Figure 5). However, compared to the countries in Southeast Asia and North Africa at lower latitudes, countries in East Asia and West Asia had fewer cases occurring in the warm/hot season from April to September (8.1% vs. 26.2%), and showed earlier peaks (December vs. January) and shorter epidemic periods, with cases occurring year round in Southeast Asia and North Africa, but from January to June and October to December in East Asia, and only from December to March in West Asia(Appendix Figure 4B and 5).

After excluding four cases with unknown outcome (two of Vietnam in 2005 and two of Egypt in 2015), the overall case fatality risk (CFR) was 53.5% (483/903), with a decrease from 70.7% (275/420) in 2003-2008 to 43.4% (202/465) in 2009-2015, and varied across geographical regions, with a CFR (69.4%, 349/503) in East and Southeast Asia more than twice that in North Africa (32.1%, 116/361) (Table 1). The age distribution of cases also differed by outcome, with a median age of 22 years (IQR: 11.5-32 years) for fatal cases and 10 years (IQR: 3-30 years) for cases who recovered (Figure 3C-D). The majority (95.8%, 748/781) of cases reported exposure to poultry including: 85.7% (439/512) exposed to sick or dead poultry; 61.4% (188/306) exposed to backyard poultry; 26.4% (82/311) exposed to LBMs; 4.7% (15/321) occupational exposure to live poultry. In addition, 6.2% (49/792) reported contact with a human H5N1 case before the onset of illness (Table 1, Appendix Table 3). Time from onset of illness to hospitalization was available for 79.7% (723/907) cases with a median of 4 days (IQR: 2-6 days). Generally, the survived cases had an earlier hospitalization than fatal cases (median and IQR: 3 and 1-6 days vs. 5 and 3-7) (Appendix Figure 6). Additionally, the cases in North Africa had a shorter time from onset to hospitalization than cases in East and Southeast Asia (median and IQR: 3 and 1-6 days vs. 5 and 3-7), but the median time from onset to outcome was the same (10 days) between cases in North Africa and cases in East and Southeast Asia.

The A(H5N1) viruses in human cases have been characterized as belonging to clade or subclade 0, 1, 2.1, 2.1, 2.3, and 7 (Table 1-2, Appendix Table 3). Clade 1 was first reported in Hong Kong SAR in 2003, and then reported in Southeast Asia each year from 2003 to 2014, but subclade 2.1 was only reported in Indonesia since 2005, and subclade 2.2 has circulated in Egypt since 2006 with sporadic reporting in Africa and West Asia. In addition, subclade 2.3 has been reported in East and Southeast Asia since 2005.

### Human cases of H5N1 in Egypt

During March 2006 – April 2015, a total of 363 human cases with influenza A(H5N1) virus infection were reported in Egypt with 116 deaths (32%) (Appendix Figure 7), of which more than half (51%) of cases were reported during the 6 months of November 2014 – April 2015 (Appendix Table 4). The male-to-female ratio was not significantly different between cases before November 2014 and cases in the period November 2014 – April 2015, but the latter had an older median age (median and IQR: 26; 4-38 years) than the former (median and IQR: 16; 3.25-30 years), which was also different for both non-fatal and fatal cases (Figure 4E-F). However, the CFR was not significantly different at 36% (64/178) before November 2014 compared to 28.4% (52/183) during November 2014 – April 2015 (Appendix Table 4). For fatal cases, the median time and IQR (5; 3-6 days) for onset to hospital admission was the same between March 2006 – October 2014 and November 2014 – April 2015, but the time was different for non-fatal cases with a median of one day (IQR: 1-3 days) before November 2014 and four days (IQR: 2-6 days) during November 2014 – April 2015. Most cases reported a history of poultry exposure - 96.1% before November 2014 and 90.8% in November 2014 – April 2015.

## DISCUSSION

In this study, a global dataset spanning 18 years was systematically collated to investigate changes in the epidemiological characteristics of human H5N1 cases, and also focused on Egypt, given its unique situation of increasing incidence since November 2014.^20,37,46^ Our analyses suggest that the geographic extent of human H5N1 cases has expanded from East Asia to Southeast Asia, then to West Asia and North Africa during 2003-2009, which may be related to the global dispersal of the virus via bird migration.^62-64^ The bird migration network was shown to better reflect the observed viral gene sequence data than other networks and contributes to seasonal H5N1 epidemics in local regions.^3,5,7^ In addition, previous evidence demonstrated Siberia as a major hub for the dispersal of the virus via bird populations, and Southeast Asia and Africa as areas of local virus persistence and the major sources of genetically and antigenically novel strains.^5,7,65,66^ Therefore, the increasing range of virus dispersal and outbreaks among birds may also increase the risk of human exposure.^3,67^ However, some of the apparent geographical dispersal in cases may also be attributed to enhanced clinical and laboratory surveillance capacity in the past 15-20 years.

Human H5N1 infections were found to exhibit seasonality, related to the cooler season from December to March and across diverse climate zones in the Northern Hemisphere (Appendix Figure 4B and 5), which may correlate with the migration patterns of wild birds and the activity of virus in winter or cooler seasons.^3,7,43^ A recent study found that the timing of H5N1 outbreaks and viral migrations were closely associated with bird migration networks in Asia.^5^ In addition, the lower temperatures in Asia and North Africa across diverse climates were associated with increasing human H5N1 virus infection in winter, which is consistent with increased poultry outbreaks and H5N1 virus transmission during cold and dry conditions, and also overlapped with human seasonal influenza epidemics.^3,43,68,69^

Although most human populations are thought to have little or no immunity to influenza A(H5N1) viruses, most cases examined in this study were children and younger adults, and these age groups were also more likely to recover, whereas the fatality risk was higher in adults, which might be related to the immunological reaction of virus in different age groups.^41^ Consistent with previous reports,^28,45^ the cases with ≥3 days from onset of illness to hospitalization were more likely to be fatal than those admitted within 3 days of onset with a odds ratios (OR) of 3.6 and 95% confidence intervals (CI) of 2.5 - 5.1, which might be due to the early administration of antiviral treatment, or selection bias where the cases admitted later after onset were more likely to be severe.^17^ Compared with Indonesia, Vietnam, Cambodia, mainland China and Thailand , the lower CFR in Egypt (Chi-squared tests, p<0.001) might be related to a less virulent virus clade, less severe clinical disease, and earlier identification, hospitalization and early treatment with oseltamivir for H5N1 cases.^20,44,70^ However, the CFR might be underestimated because various government entities or reports may not have identified or updated which cases have died at the time we collated data. Additionally, almost all human cases of H5N1 infection had a recent history of close contact with infected live or dead birds, other human cases, or H5N1-contaminated environments, which reaffirmed reports that human H5N1 virus infection is typically preceded by exposure to sick or dead poultry in backyards, LBMs or farms.^71-76^

An increased number of animal-to-human infections has been reported by Egypt during November 2014 – April 2015 with the number of cases more than the total of the last 8 years from 2006-2014.^20^ The increase in the number of human cases in Egypt since November 2014 can be attributed to a mixture of factors, including increased circulation of H5N1 viruses in poultry, lower public health awareness of risks in middle and upper Egypt and seasonal factors, such as closer proximity to poultry because of cold weather and possible longer survival of the viruses in the environment.^77^ However, the increased numbers of human cases in Egypt are of major concern because of the continued potential pandemic threat from H5N1. A few cases of human-to-human transmission and family clusters have been reported in Egypt and other countries.^40,46,78-82^ Nevertheless, H5N1 viruses do not currently appear to transmit easily from person to person, and the risk of community level spread of these viruses remains low.^20,27,39^

H5N1 viruses have evolved from the 1996 progenitor strain and now comprise at least 10 clades, through a complexity of genetic changes, which have infected domestic poultry and wild birds in many countries.^21,62,63,83,84^ In this study, 4 clades (0, 1, 2, and 7) and 3 subclades (2.1, 2.2, and 2.3) of H5N1 virus strains have infected humans, all of which have been reported in human cases before 2006.^41,85^ Compared to clade 0, the cases with clade 1, subclade 2.1 and 2.3 were more likely to result in death with a crude OR of 2.8 (95% CI: 0.93, 9.6), 11.0 (95%CI: 3.5, 37.8) and 3.2 (95%CI: 1.0, 11.4) respectively (Appendix Table 3). However, the risk of death between cases with clade 0 and subclade 2.1 was not significantly different (OR: 1.0; 95% CI: 0.3, 3.3). Based on available information, the clades of viruses isolated from humans were the same as the clades circulating in local poultry.^21,28^ During the period from late 2003 to mid-2005, most H5N1 virus infections in humans were caused by clade 1 strains in Southeast Asia (i.e., Vietnam, Thailand, and Cambodia).^85^

Although the highly pathogenic H5N1 virus strains can be expected to continue evolving over time, preliminary laboratory investigation has not detected major genetic changes in the viruses isolated from the patients or animals in 2014-2015 compared to previously circulating isolates in the same regions,^41,86^ and the genetic diversity of the H5N1 virus decreased significantly between 1996 and 2011 in China, presumably under strong selective pressure associated with vaccination in poultry.^56^ However, other influenza A(H5) subtypes, such as influenza A(H5N2), A(H5N3), A(H5N6) and A(H5N8), have recently been detected in birds in Europe, North America, and Asia, and so far no human cases of infection have been reported, with the exception of three human infections with influenza A(H5N6) virus detected in China in 2014-15.^39,77^ However, the co-circulation of influenza A viruses in human and animal reservoirs can provide opportunities for these viruses to re-assort and acquire the genetic characteristics that facilitate sustained human-to-human transmission, a necessary trait of pandemic viruses.^3,87^

Vaccines and antivirals are the most effective approaches to prevent influenza virus infection and treat illness respectively.^41,88,89^ Vaccination of poultry has been implemented in many of the affected countries to control H5N1 in poultry, especially in those locations where H5N1 viruses have become enzootic in poultry and wild birds.^90-92^ Currently, 27 A(H5N1) candidate vaccine viruses for humans are available and a new candidate vaccine is in preparation to protect against the currently circulating H5 clade 2.2.1.2 of viruses, covering all the recent H5N1 virus isolates from Egypt.^41,93^ The first adjuvant vaccine for the prevention of H5N1 influenza has been approved by the United States Food and Drug Administration in November 2013, and this vaccine is being stockpiled for pandemic preparedness by the United States government.^94^ In addition, the antiviral drug oseltamivir can reduce the severity of illness and mortality when started soon after symptom onset and appears to benefit all age groups. Prompt diagnosis and early therapeutic intervention should therefore be considered for all H5N1 cases,^89,95,96^ though antiviral resistance continues to receive attention and there is a need for continued monitoring.^97^ The availability of antivirals and vaccines in the event of a H5N1 pandemic should be considered in advance.^98^

There are some limitations to this study. First, the data used were collated from different sources. The data quality may be influenced by key steps in public health surveillance or reports including the case definitions, reporting methods, availability of health care and laboratory diagnostics, under reporting, and the completeness and accuracy of data reported or announced by different countries or organizations. Compared to the areas where many cases were seen in this study, some countries with few cases or without cases reported might be attributed to the low availability and capability of public health services, serological testing, and surveillance. Second, detailed data on case characteristics and clinical management were unavailable to assess the association between clinical manifestation, treatment and outcome, and this study did not include the cases with subclinical H5N1 virus infection, which have been occasionally reported.^72,99-101^ Third, the findings might be influenced by missing data on exposure, outcome and hospitalization, and the misclassification of cases with presumed clade or subclade. In addition, this study only included data sources in English or Chinese, which might neglect data on cases reported in other languages, including announcements or reports from Egypt.

In conclusion, the high-risk areas, population groups and seasonality of human HPAI H5N1 infections have been systematically reviewed here, providing evidence for the planning of prevention and control. The geographic distribution of countries with human H5N1 infections has expanded, especially between 2003 and 2008, with variations in outcome, demography, seasonality and the clade or subclade of viruses across the region. The incidence of human infections increased dramatically in Egypt from November 2014 to April 2015, but remained at a low level in other regions, and the CFR in Egypt has not significantly changed. However, since avian influenza A(H5N1) viruses present a continuous threat to human populations, echoing the recommendations of WHO and other organizations on influenza at the human-animal interface,^41,89,102-104^ there is a need for sustained efforts and close collaboration between the animal health and public heath sectors at community, national, and international levels to monitor the dynamics in human, poultry and wild birds, and to conduct early clinical management, while downstream research is encouraged to develop vaccines and antivirals, explore the driving factors behind the epidemic, and evaluate the potential for future pandemics.

## Supplementary Material

1 **Appendix Table 1** The data source of human case with H5N1 virus infection in each country, May 1997 – April 2015.
**Appendix Table 2** The list of variables in the individual dataset of human case with H5N1 virus infection, May 1997 – April 2015.
**Appendix Table 3** Demographic and Epidemiologic characteristics of human case with H5N1 virus infection by outcomes, May 1997 – April 2015.
**Appendix Table 4** The characteristics of human case with H5N1 virus infection in Egypt before and since 1 November 2014.
**Appendix Figure 1** Epidemic curve of human cases with H5N1 virus infection by climate zones, May 1997 – April 2015.
**Appendix Figure 2** The number of human cases with H5N1 virus infection by year and geographic region, May 1997 – April 2015 (N=907).
**Appendix Figure 3** The number of human cases with H5N1 virus infection by year and country, May 1997 – April 2015 (N=907).
**Appendix Figure 4** Heat map of the reported data of human cases with H5N1 virus infection by country, sorted by geographical region and the date of the first cases illness onset, May 1997–April 2015.
**Appendix Figure 5** The seasonality of human cases with H5N1 virus infection by the month of illness onset, May 1997 – April 2015.
**Appendix Figure 6** The distribution of days from onset to hospital admission of human H5N1 cases by outcome and geographic region, May 1997–April 2015.
**Appendix Figure 7** The geographic distribution of human cases with H5N1 virus infection by outcome in Egypt, March 2006–April 2015 (n=363).

## Figures and Tables

**Table 1:** The characteristics of human case with H5N1 virus infection by geographic region, May 1997 – April 2015

Characteristics	Total (n=907)	East and Southeast Asia (n=505)	North Africa (n=363)	Other (n=39)
Type of cases				

Confirmed case	858 (94.6%)	479 (94.9%)	343 (94.5%)	36 (92.3%)
Probable case	49 (5.4%)	26 (5.1%)	20 (5.5%)	3 (7.7%)

Sex				

Female	476 (52.5%)	246 (48.7%)	213 (58.7%)	17 (43.6%)
Unknown	29 (3.2%)	21 (4.2%)	6 (1.7%)	2 (5.1%)

Age				

Median (yrs, range)	19 (0.25, 86)	19 (0.3, 75)	20 (0.25, 86)	15 (1.3, 52)

Final outcome				

Death	483 (53.3%)	349 (69.1%)	116 (32%)	18 (46.2%)
Unknown	4 (0.4%)	2 (0.4%)	2 (0.6%)	0 (0)

Hospitalization				

Yes	819 (90.3%)	438 (86.7%)	353 (97.2%)	28 (71.8%)
Unknown	82 (9%)	64 (12.7%)	9 (2.5%)	9 (23.1%)

Median of time delay (days, range)				

Time from onset to hospital admission	4 (0, 90)	5 (0, 90)	3 (0, 33)	2 (0, 13)
Unknown	184 (20.3%)	121 (24%)	46 (12.7%)	17 (43.6%)
Time from hospital admission to death or discharge (recovery)	5 (0, 116)	4 (0, 116)	5 (0, 28)	5 (2, 25)
Unknown	403 (44.4%)	166 (32.9%)	219 (60.3%)	18 (46.2%)
Time from onset to death or discharge (recovery)	10 (0, 119)	10 (0, 119)	10 (2, 32)	9 (2, 32)
Unknown	360 (39.7%)	124 (24.6%)	221 (60.9%)	15 (38.5%)

Predominant clade or subclade				

0	18 (2%)	18 (3.6%)	0 (0)	0 (0)
1	193 (21.3%)	193 (38.2%)	0 (0)	0 (0)
2.1	208 (22.9%)	208 (41.2%)	0 (0)	0 (0)
2.2	393 (43.3%)	0 (0)	363 (100%)	30 (76.9%)
2.3	89 (9.8%)	84 (16.6%)	0 (0)	5 (12.8%)
7	2 (0.2%)	2 (0.4%)	0 (0)	0 (0)
Unknown	4 (0.4%)	0 (0)	0 (0)	4 (10.3%)

Exposure history				

Any exposure to poultry	748 (82.5%)	382 (75.6%)	339 (93.4%)	27 (69.2%)
Unknown	126 (13.9%)	94 (18.6%)	24 (6.6%)	8 (20.5%)
Occupational exposure to live poultry	15 (1.7%)	12 (2.4%)	2 (0.6%)	1 (2.6%)
Unknown	586 (64.6%)	289 (57.2%)	286 (78.8%)	11 (28.2%)
Visit LBMs	82 (9%)	68 (13.5%)	11 (3%)	3 (7.7%)
Unknown	596 (65.7%)	296 (58.6%)	286 (78.8%)	14 (35.9%)
Exposure to sick or dead poultry	439 (48.4%)	242 (47.9%)	174 (47.9%)	23 (59%)
Unknown	395 (43.6%)	217 (43%)	166 (45.7%)	12 (30.8%)
Exposure to backyard poultry	188 (20.7%)	113 (22.4%)	64 (17.6%)	11 (28.2%)
Unknown	601 (66.3%)	301 (59.6%)	286 (78.8%)	14 (35.9%)
Human case contact	49 (5.4%)	35 (6.9%)	3 (0.8%)	11 (28.2%)
Unknown	115 (12.7%)	86 (17%)	21 (5.8%)	8 (20.5%)

Note: Data are presented as no. (%) of patients unless otherwise indicated. LBMs: Live bird markets. East and Southeast Asia (505 cases): Indonesia (208), Viet Nam (134), Cambodia (58), mainland China (52), Thailand (27), Hong Kong SAR (23), Laos (2), and Myanmar (1); North Africa (363 cases): Egypt (363); Other (39 cases): Turkey (12), Azerbaijan (9), Bangladesh (7), Pakistan (4), Iraq (3), Nigeria (2), Djibouti (1), and Canada (1). Data on H5N1 clade or subclade of Human cases was based on the reports from WHO website, or the literature, and the known geographic distribution of the viruses. No all cases were laboratory confirmed and reported with clade results, so we presumed that the case in each area was infected by the reported predominant clade or subclade of H5N1 virus in the same period and area. The clade or subclade in each area were clade 0 in Hong Kong SAR in 1997, clade 1 in Viet Nam, Cambodia, Thailand, and Hong Kong SAR, subclade 2.1 mainly in Indonesia, 2.2 in Egypt, Turkey, Azerbaijan, Bangladesh, Iraq, Nigeria and Djibouti, and 2.3 in Viet Nam, Viet Nam, Bangladesh, Laos, Canada and Myanmar, and 7 in mainland China. The data of clade was unavailable for 4 cases in Pakistan in 2007.

**Table 2:** The clade or subclade and fatality of human case with H5N1 virus infection, May 1997 – April 2015

Clade or subclade	Year first identified	Locations identified	Case fatality risk
0	1997	Hong Kong SAR	31.6% (6/18)
1	2003	Hong Kong SAR, Vietnam, Cambodia and Thailand	58.6% (112/191)
2.1	2005	Indonesia	84.6% (176/208)
2.2	2005	Turkey, Egypt, Azerbaijan, Djibouti, Iraq, Nigeria, and Bangladesh	33.2% (130/391)
2.3	2005	Mainland China, Laos, Myanmar, Vietnam, Hong Kong SAR, Bangladesh and Canada	61.8% (55/89)
7	2003	Mainland China	100% (2/2)

Note: Data on H5N1 clade or subclade of Human cases was based on the reports from WHO website, or the literature, and the known geographic distribution of the viruses. No all cases were laboratory confirmed and reported with clade results, so we presumed that the case was infected by the reported clade or subclade of H5N1 virus in the same period and area. The data of clade was unavailable for four cases in Pakistan in 2007, and four cases with unknown outcome (two of Viet Nam in 2005 and two of Egypt in 2015) were also excluded.

## Box 1

Definition of exposures to poultry and humans

**Type of exposure**	**Definition**

***Occupational exposure to live poultry***	refers to poultry related professional exposure (e.g. poultry breeding, trafficking, sale, and quarantine) in the two weeks prior to the onset of illness.

***Visiting live bird market (LBM)***	refers to the visit of a wholesale or retail market of live poultry or birds within the two weeks prior to the onset of symptoms.

***Exposure to sick or dead poultry***	refers to direct physical contact with, or in proximity to, sick or dead poultry or its production or faeces in the two weeks prior to onset.

***Exposure to backyard poultry***	refers to whether the case had been exposed to poultry raised in the backyard within two weeks prior to onset.

***Any exposure to poultry***	refers to direct contact, indirect contact or proximity to healthy, sick or dead poultry (including any kind of poultry or birds, e.g. chicken, ducks, goose, pet birds, pigeons, et al.) in LBMs, backyard, farm, or neighborhood, or consumption of improperly processed poultry products.

***Human case contact***	refers to a patient with a history of close contact with a confirmed or probable human H5N1 case (any time from the day before the onset of illness to death or during the period that case was hospitalized) in the two weeks before the onset of symptoms.
