# Supplementary material for: Global epidemiology of avian influenza A(H5N1) virus infection in humans, 1997 – 2015: a systematic review
Source: Lancet Infect Dis. Author manuscript; Available in PMC 2017 Jul 1. (PMC4933299; doi:10.1016/S1473-3099(16)00153-5)
Supplement: 1 — Appendix Table 1 The data source of human case with H5N1 virus infection in each country, May 1997 – April 2015. Appendix Table 2 The list of variables in the individual dataset of human case with H5N1 virus infection, May 1997 – April 2015. Appendix Table 3 Demographic and Epidemiologic characteristics of human case with H5N1 virus infection by outcomes, May 1997 – April 2015. Appendix Table 4 The characteristics of human case with H5N1 virus infection in Egypt before and since 1 November 2014. Appendix Figure 1 Epidemic curve of human cases with H5N1 virus infection by climate zones, May 1997 – April 2015. Appendix Figure 2 The number of human cases with H5N1 virus infection by year and geographic region, May 1997 – April 2015 (N=907). Appendix Figure 3 The number of human cases with H5N1 virus infection by year and country, May 1997 – April 2015 (N=907). Appendix Figure 4 Heat map of the reported data of human cases with H5N1 virus infection by country, sorted by geographical region and the date of the first cases illness onset, May 1997–April 2015. Appendix Figure 5 The seasonality of human cases with H5N1 virus infection by the month of illness onset, May 1997 – April 2015. Appendix Figure 6 The distribution of days from onset to hospital admission of human H5N1 cases by outcome and geographic region, May 1997–April 2015. Appendix Figure 7 The geographic distribution of human cases with H5N1 virus infection by outcome in Egypt, March 2006–April 2015 (n=363). [file NIHMS789852-supplement-1.docx]

**Supporting Information**

**Appendix Table 1. The data source of human case with H5N1 virus infection by country, May 1997 – April 2015**

| **Country/Area** | **The initial data source of case lists** | **Main sources for extracting data of individual case** |
| --- | --- | --- |
| Mainland China | 50 confirmed cases  – from WHO and China CDC  2 probable cases  – from China CDC and literature in PubMed | 1. Chinese Center for Disease Control and Prevention. National Notifiable Infectious Disease Reporting Information System. <http://1.202.129.170> 2. WHO. Monthly Risk Assessment Summary: Influenza at the Human-Animal Interface. 2011-2015. 3. H5N1 Human Case Lists 2012-2015. Flu Trackers. <https://flutrackers.com/> 4. Yu H, et al. Lancet,2006,367(9504):84. 5. Wang H, et al. Lancet,2008,371(9622):1427-1434. |
| Hong Kong SAR, China | 20 confirmed cases  – 18 cases in 1997 from MMWR of US CDC  – other two cases from WHO  3 probable cases  – from literature in PubMed | 1. Centre for Health Protection, Hong Kong SAR. Avian Influenza Report. <http://www.chp.gov.hk/en/guideline1_year/29/134/332.html> 2. Center for Disease Control and Prevention. Morbidity and Mortality Weekly Report (MMWR). Two reports in 19 Dec 1997 and 9 Jan 1998. 3. WHO. Weekly Epidemiological Record (WER) in 72(50), 72(51), 73(1), 73(3), 73(4), 73(5) and 73(12). 4. WHO. Monthly Risk Assessment Summary: Influenza at the Human-Animal Interface. 2011-2015. 5. Post in ProMED-mail. <http://www.promedmail.org/> 6. Peiris JS, et al. Lancet, 2004. 363(9409): 617-619. |
| Vietnam | 127 confirmed cases  – from WHO and Vietnam National Institute of Hygiene and Epidemiology  7 probable cases  – from Vietnam National Institute of Hygiene and Epidemiology and literature in PubMed | 1. Vietnam National Institute of Hygiene and Epidemiology 2. WHO. Monthly Risk Assessment Summary: Influenza at the Human-Animal Interface. 2011-2015. 3. WHO Western Pacific Region. Avian Influenza Weekly Update. <http://www.wpro.who.int/emerging_diseases/AvianInfluenza/en/> 4. H5N1 Human Case Lists 2012-2015. Flu Trackers. <https://flutrackers.com/> 5. Post in ProMED-mail. <http://www.promedmail.org/> 6. Tran TH, et al. N Engl J Med,2004,350(12):1179-1188. 7. Menno D, et al. N Engl J Med,2005,352(7):686-691. |
| Thailand | 25 confirmed cases   - from WHO   2 probable cases  – from literature in PubMed | 1. WHO. Weekly Epidemiological Record (WER) in 79(07), 83(40). 2. WHO Western Pacific Region. Avian Influenza Weekly Update. <http://www.wpro.who.int/emerging_diseases/AvianInfluenza/en/> 3. Ungchusak K, et al. N Engl J Med,2005,352(4):333-340. 4. Olsen SJ, et al. Emerg Infect Dis 2005; 11(11):1799-801. |
| Cambodia | 56 confirmed cases  – from WHO  2 probable cases  – from the weekly report of WHO Western Pacific Region and literature in PubMed | 1. WHO. Disease Outbreak News of the Global Alert and Response 2. WHO. Monthly Risk Assessment Summary: Influenza at the Human-Animal Interface. 2011-2015. 3. WHO Western Pacific Region. Avian Influenza Weekly Update. Reports in 7, 14, and 21 February 2014. <http://www.wpro.who.int/emerging_diseases/AvianInfluenza/en/> 4. H5N1 Human Case Lists 2012-2015. Flu Trackers. <https://flutrackers.com/> 5. Olsen SJ, et al. Emerg Infect Dis, 2005, 11(11): 1799-1801. |
| Indonesia | 199 confirmed cases  – from WHO  9 probable cases  – from the weekly report of WHO Western Pacific Region and literature in PubMed | 1. WHO. Monthly Risk Assessment Summary: Influenza at the Human-Animal Interface. 2011-2015. 2. WHO Western Pacific Region. Avian Influenza Weekly Update. <http://www.wpro.who.int/emerging_diseases/AvianInfluenza/en/> 3. WHO. Disease Outbreak News of the Global Alert and Response 4. WHO. Weekly Epidemiological Record (WER). 5. H5N1 Human Case Lists 2012-2015. Flu Trackers. <https://flutrackers.com/> 6. Post in ProMED-mail. <http://www.promedmail.org/> 7. Kandun IN, et al. N Engl J Med,2006,355(21):2186-2194. 8. Yang Y, et al. Emerg Infect Dis,2007,13(9):1348-1353. 9. Olsen SJ, et al. Emerg Infect Dis, 2005, 11(11): 1799-1801. |
| Laos | 2 confirmed cases  – from WHO | 1. WHO. Weekly Epidemiological Record (WER). 2. WHO Western Pacific Region. Avian Influenza Weekly Update. <http://www.wpro.who.int/emerging_diseases/AvianInfluenza/en/> |
| Myanmar | 1 confirmed cases  – from WHO | 1. WHO. Weekly Epidemiological Record (WER). 2. WHO Western Pacific Region. Avian Influenza Weekly Update. <http://www.wpro.who.int/emerging_diseases/AvianInfluenza/en/> |
| Egypt | 342 confirmed cases  – from WHO  21 probable cases  – from the case lists of FluTrackers with the announcements from health authorities. | 1. WHO. Monthly Risk Assessment Summary: Influenza at the Human-Animal Interface. 2011-2015. 2. WHO. Disease Outbreak News of the Global Alert and Response 3. WHO. Weekly Epidemiological Record (WER). 4. H5N1 Human Case Lists 2012-2015. Flu Trackers. <https://flutrackers.com/> 5. Post in ProMED-mail. <http://www.promedmail.org/> 6. Kandeel A, et al. Emerg Infect Dis,2010,16(7):1101-1107. |
| Turkey | 12 confirmed cases  – from WHO and literature in PubMed | 1. WHO. Weekly Epidemiological Record (WER), 81(02), 81(03), 81(05), and 81(43), etc. 2. Oner AF, et al. N Engl J Med,2006,355(21):2179-2185. |
| Iraq | 3 confirmed cases  – from WHO | 1. WHO. Monthly Risk Assessment Summary: Influenza at the Human-Animal Interface. 2011-2015. 2. WHO. Disease Outbreak News of the Global Alert and Response 3. WHO. Weekly Epidemiological Record (WER). |
| Azerbaijan | 8 confirmed cases  – from WHO and Azerbaijan Ministry of Health  1 probable cases  – from Azerbaijan Ministry of Health. | 1. Azerbaijan Ministry of Health. 2. WHO. Weekly Epidemiological Record (WER). Two reports in 2006, 81(12) and 2006, 81(18). 3. Gilsdorf A, et al. Euro Surveillance, 2006, 11(5): 122-126. |
| Djibouti | 1 confirmed cases  – from WHO | 1. WHO. Disease Outbreak News of the Global Alert and Response 2. H5N1 Human Case Lists 2012-2015. Flu Trackers. <https://flutrackers.com/> |
| Nigeria | 1 confirmed cases  1 probable cases  – from WHO | 1. WHO. Disease Outbreak News of the Global Alert and Response 2. Post in ProMED-mail. <http://www.promedmail.org/> 3. WHO. Weekly Epidemiological Record (WER). |
| Pakistan | 3 confirmed cases  1 probable cases  – from WHO | 1. WHO. Weekly Epidemiological Record (WER), 83(40). 2. WHO. Disease Outbreak News of the Global Alert and Response. <http://www.who.int/csr/don/2007_12_27/en/> |
| Bangladesh | 7 confirmed cases  – from WHO | 1. WHO. Monthly Risk Assessment Summary: Influenza at the Human-Animal Interface. 2011-2015. 2. H5N1 Human Case Lists 2012-2015. Flu Trackers. <https://flutrackers.com/> 3. Post in ProMED-mail. <http://www.promedmail.org/> 4. Brooks WA, et al. Emerg Infect Dis,2009,15(8):1311-1313. |
| Canada | 1 confirmed cases  – from WHO | 1. WHO. Monthly Risk Assessment Summary: Influenza at the Human-Animal Interface. 2. Post in ProMED-mail on 12 Jan 2014. <http://www.promedmail.org/> 3. Two reports in Center for Infectious Disease Research and Policy (CIDRAP) on 8 Jan 2014 and 14 Jan 2014. |

**Appendix Table 2. The list of variables in the individual dataset of human case with H5N1 virus infection****, May 1997 – April 2015**

| **Variables** | **Definition/classification** | **Completeness**  **(n=907)** |
| --- | --- | --- |
| Gender | Male and Female | 97.13% |
| Age | The interval time from the date of birth to the date of onset | 96.80% |
| Country | The living country of case when the case was recorded. | 100% |
| Type of diagnosis | Probable case and confirmed case | 100% |
| Year of onset | The year of illness onset | 100% |
| Month of onset | The month of illness onset | 97.46% |
| Week of onset | The week of illness onset | 95.26% |
| Date of onset | The date of illness onset | 91.73% |
| Hospitalization | Admission to hospital or No | 90.96% |
| Date of hospitalization | The data of admission to hospital | 81.81% |
| Outcome | Fatal or non-fatal (recovery/alive when the data was collated as of 1 May 2015) | 99.56% |
| Date of outcome | The date of death or discharge from hospital or alive when the case was reported | 66.04% |
| Predominant clade or subclade | See the definition in the Methods. | 99.56% |
| Any exposure to poultry | Yes or No | 86.11% |
| Occupational exposure to live poultry | Yes or No | 35.39% |
| Visit LBMs | Yes or No | 34.29% |
| Exposure to sick or dead poultry | Yes or No | 56.45% |
| Exposure to backyard poultry | Yes or No | 33.74% |
| Human case contact | Yes or No | 87.32% |
| Time from onset to admission | The lag days from illness onset to hospital admission | 79.71% |
| Time from admission to outcome | The lag days from hospital admission to death or recovery or alive when the case was reported) | 55.57% |
| Time from onset to outcome | The lag days from illness onset to death or recovery or alive when the case was reported) | 60.31% |

**Appendix Table 3. Demographic and Epidemiologic characteristics of human case with H5N1 virus infection by outcomes, May 1997 – April 2015**

| Characteristics | Total (n=903) | Fatal cases (n=483) | Non-fatal cases (n=420) | Odds ratio (95% CI) | p value |
| --- | --- | --- | --- | --- | --- |
| Type of cases | | | | | |
| Confirmed case | 856 (94.8%) | 452 (93.6%) | 404 (96.2%) | - | - |
| Probable case | 47 (5.2%) | 31 (6.4%) | 16 (3.8%) | - | - |
| Sex | | | | | |
| Female | 474 (52.5%) | 263 (54.5%) | 211 (50.2%) | Ref | - |
| Male | 402 (44.5%) | 201 (41.6%) | 201 (47.9%) | 1.25 (0.95, 1.64) | 0.105 |
| Unknown | 27 (3%) | 19 (3.9%) | 8 (1.9%) | - | - |
| Age | | | | | |
| Median (yrs, range) | 19 (0.25, 86) | 22 (1, 77) | 10 (0.25, 86) | - | <0.001 |
| Age group |  |  |  |  |  |
| 0-4 | 189 (20.9%) | 45 (9.3%) | 144 (34.3%) | Ref | - |
| 5-14 | 173 (19.2%) | 97 (20.1%) | 76 (18.1%) | 4.08 (2.14, 6.58) | <0.001 |
| 15-24 | 163 (18.1%) | 111 (23%) | 52 (12.4%) | 6.83 (4.16, 11.24) | <0.001 |
| 25-34 | 180 (19.9%) | 117 (24.2%) | 63 (15%) | 5.94 (3.69, 9.61) | <0.001 |
| 35-44 | 109 (12.1%) | 64 (13.3%) | 45 (10.7%) | 4.55 (2.66, 7.80) | <0.001 |
| 45-54 | 37 (4.1%) | 18 (3.7%) | 19 (4.5%) | 3.03 (1.36, 6.66) | 0.002 |
| 55-64 | 17 (1.9%) | 6 (1.2%) | 11 (2.6%) | 1.75 (0.50, 5.49) | 0.293 |
| 65 and above | 11 (1.2%) | 5 (1%) | 6 (1.4%) | 2.67 (0.61, 10.98) | 0.107 |
| Unknown | 24 (2.7%) | 20 (4.1%) | 4 (1%) | - | - |
| Hospitalization | | | | | |
| Yes | 817 (90.5%) | 424 (87.8%) | 393 (93.6%) | - | - |
| No | 6 (0.7%) | 2 (0.4%) | 4 (1%) | - | - |
| Unknown | 80 (8.9%) | 57 (11.8%) | 23 (5.5%) | - | - |
| Country | | | | | |
| Egypt | 361 (40%) | 116 (24%) | 245 (58.3%) | Ref | - |
| Indonesia | 208 (23%) | 176 (36.4%) | 32 (7.6%) | 11.62 (7.38, 18.54) | <0.001 |
| Vietnam | 132 (14.6%) | 70 (14.5%) | 62 (14.8%) | 2.38 (1.55, 3.66) | <0.001 |
| Cambodia | 58 (6.4%) | 39 (8.1%) | 19 (4.5%) | 4.34 (2.32, 8.28) | <0.001 |
| Mainland China | 52 (5.8%) | 35 (7.2%) | 17 (4%) | 4.35 (2.25, 8.61) | <0.001 |
| Thailand | 27 (3%) | 19 (3.9%) | 8 (1.9%) | 5.02 (2.02, 13.59) | <0.001 |
| Hong Kong, China | 23 (2.5%) | 8 (1.7%) | 15 (3.6%) | 1.13 (0.40, 2.93) | 0.792 |
| Turkey | 12 (1.3%) | 4 (0.8%) | 8 (1.9%) | 1.06 (0.23, 4.04) | 0.93 |
| Azerbaijan | 9 (1%) | 6 (1.2%) | 3 (0.7%) | 4.22 (0.88, 26.44) | 0.029 |
| Bangladesh | 7 (0.8%) | 1 (0.2%) | 6 (1.4%) | - | - |
| Pakistan | 4 (0.4%) | 2 (0.4%) | 2 (0.5%) | - | - |
| Iraq | 3 (0.3%) | 2 (0.4%) | 1 (0.2%) | - | - |
| Laos | 2 (0.2%) | 2 (0.4%) | 0 (0) | - | - |
| Nigeria | 2 (0.2%) | 2 (0.4%) | 0 (0) | - | - |
| Myanmar | 1 (0.1%) | 0 (0) | 1 (0.2%) | - | - |
| Djibouti | 1 (0.1%) | 0 (0) | 1 (0.2%) | - | - |
| Canada | 1 (0.1%) | 1 (0.2%) | 0 (0) | - | - |
| Year of onset | | | | | |
| 1997 | 18 (2%) | 6 (1.2%) | 12 (2.9%) | Ref | - |
| 2003 | 8 (0.9%) | 7 (1.4%) | 1 (0.2%) | 14.0 (1.19, 678.91) | 0.011 |
| 2004 | 52 (5.8%) | 40 (8.3%) | 12 (2.9%) | 6.67 (1.8, 25.93 ) | 0.001 |
| 2005 | 107 (11.8%) | 49 (10.1%) | 58 (13.8%) | 1.67 (0.54, 5.89) | 0.324 |
| 2006 | 115 (12.7%) | 82 (17%) | 33 (7.9%) | 4.97 (1.55, 17.32) | 0.002 |
| 2007 | 91 (10.1%) | 62 (12.8%) | 29 (6.9%) | 4.28 (1.31, 15.13) | 0.005 |
| 2008 | 47 (5.2%) | 35 (7.2%) | 12 (2.9%) | 5.83 (1.56, 22.86) | 0.002 |
| 2009 | 73 (8.1%) | 32 (6.6%) | 41 (9.8%) | 1.56 (0.48, 5.62) | 0.418 |
| 2010 | 48 (5.3%) | 24 (5%) | 24 (5.7%) | 2.0 (0.57, 7.55) | 0.226 |
| 2011 | 62 (6.9%) | 34 (7%) | 28 (6.7%) | 2.43 (0.72, 8.86) | 0.108 |
| 2012 | 32 (3.5%) | 20 (4.1%) | 12 (2.9%) | 3.33 (0.86, 13.63) | 0.048 |
| 2013 | 39 (4.3%) | 25 (5.2%) | 14 (3.3%) | 3.57 (0.96, 14.06) | 0.03 |
| 2014 | 61 (6.8%) | 27 (5.6%) | 34 (8.1%) | 1.59 (0.47, 5.83) | 0.409 |
| 2015 | 150 (16.6%) | 40 (8.3%) | 110 (26.2%) | 0.73 (0.23, 2.53) | 0.549 |
| Month of onset | | | | | |
| Jan-Mar | 484 (53.6%) | 230 (47.6%) | 254 (60.5%) | Ref | - |
| Apr-Jun | 137 (15.2%) | 75 (15.5%) | 62 (14.8%) | 1.34 (0.9, 1.99) | 0.135 |
| Jul-Sep | 80 (8.9%) | 55 (11.4%) | 25 (6%) | 2.43 (1.43, 4.2) | <0.001 |
| Oct-Dec | 179 (19.8%) | 104 (21.5%) | 75 (17.9%) | 1.53 (1.07, 2.2) | 0.016 |
| Unknown | 23 (2.5%) | 19 (3.9%) | 4 (1%) | - | - |
| Median of time delay (days, range) | | | | | |
| From onset to hospital admission | 4 (0, 90) | 5 (0, 20) | 3 (0, 90) | - | <0.001 |
| <3 days | 205 (22.7%) | 63 (13%) | 142 (33.8%) | Ref | - |
| ≥3 days | 518 (57.4%) | 317 (65.6%) | 201 (47.9%) | 3.55 (2.48, 5.11) | <0.001 |
| Unknown | 180 (19.9%) | 103 (21.3%) | 77 (18.3%) | - | - |
| From admission to outcome | 5 (0, 116) | 3 (0, 85) | 13 (0, 116) | - | - |
| Unknown | 399 (44.2%) | 103 (21.3%) | 296 (70.5%) | - | - |
| From onset to outcome | 10 (0, 119) | 9 (0, 88) | 17 (3, 119) | - | - |
| Unknown | 356 (39.4%) | 67 (13.9%) | 289 (68.8%) | - | - |
| Clade or subclade | | | | | |
| 0 | 18 (2%) | 6 (1.2%) | 12 (2.9%) | Ref | - |
| 1 | 191 (21.2%) | 112 (23.2%) | 79 (18.8%) | 2.84 (0.93, 9.57) | 0.038 |
| 2.1 | 208 (23%) | 176 (36.4%) | 32 (7.6%) | 11.0 (3.47, 37.83) | <0.001 |
| 2.2 | 391 (43.3%) | 130 (26.9%) | 261 (62.1%) | 1.0 (0.34, 3.31) | 0.994 |
| 2.3 | 89 (9.9%) | 55 (11.4%) | 34 (8.1%) | 3.24 (1.0, 11.41) | 0.026 |
| 7 | 2 (0.2%) | 2 (0.4%) | 0 (0) | - | - |
| Unknown | 4 (0.4%) | 2 (0.4%) | 2 (0.5%) | - | - |
| Exposure history | | | | | |
| Any exposure to poultry | | | | | |
| No | 33 (3.7%) | 19 (3.9%) | 14 (3.3%) | Ref | - |
| Yes | 746 (82.6%) | 379 (78.5%) | 367 (87.4%) | 0.76 (0.35, 1.63) | 0.446 |
| Unknown | 124 (13.7%) | 85 (17.6%) | 39 (9.3%) | - | - |
| Occupational exposure to live poultry | | | | | |
| No | 306 (33.9%) | 183 (37.9%) | 123 (29.3%) | Ref | - |
| Yes | 15 (1.7%) | 10 (2.1%) | 5 (1.2%) | 1.34 (0.41, 5.13) | 0.596 |
| Unknown | 582 (64.5%) | 290 (60%) | 292 (69.5%) | - | - |
| Visit LBMs | | | | | |
| No | 229 (25.4%) | 133 (27.5%) | 96 (22.9%) | Ref | - |
| Yes | 82 (9.1%) | 51 (10.6%) | 31 (7.4%) | 1.19 (0.69, 2.07) | 0.515 |
| Unknown | 592 (65.6%) | 299 (61.9%) | 293 (69.8%) | - | - |
| Exposure to sick or dead poultry | | | | | |
| No | 73 (8.1%) | 44 (9.1%) | 29 (6.9%) | Ref | - |
| Yes | 439 (48.6%) | 247 (51.1%) | 192 (45.7%) | 0.85 (0.49, 1.44) | 0.522 |
| Unknown | 391 (43.3%) | 192 (39.8%) | 199 (47.4%) | - | - |
| Exposure to backyard poultry |  |  |  |  |  |
| No | 118 (13.1%) | 67 (13.9%) | 51 (12.1%) | Ref | - |
| Yes | 188 (20.8%) | 111 (23%) | 77 (18.3%) | 1.1 (0.67, 1.79) | 0.696 |
| Unknown | 597 (66.1%) | 305 (63.1%) | 292 (69.5%) | - | - |
| Human case contact | | | | | |
| No | 741 (82.1%) | 377 (78.1%) | 364 (86.7%) | Ref | - |
| Yes | 49 (5.4%) | 27 (5.6%) | 22 (5.2%) | 1.18 (0.64, 2.23) | 0.567 |
| Unknown | 113 (12.5%) | 79 (16.4%) | 34 (8.1%) | - | - |

Note: Data are presented as no. (%) of patients unless otherwise indicated. LBMs: Live bird markets. Four cases with unknown outcome (two of Vietnam in 2005 and two of Egypt in 2015) were excluded from this table. Data on H5N1 clade or subclade of Human cases was based on the reports from WHO website, or the literature, and the known geographic distribution of the viruses. No all cases were laboratory confirmed and reported with clade results, so we presumed that the case was infected by the reported clade or subclade of H5N1 virus in the same period and area. The predominate clade or subclade in each area were clade 0 in Hong Kong SAR in 1997, clade 1 in Vietnam, Cambodia, Thailand, and Hong Kong SAR, subclade 2.1 mainly in Indonesia, 2.2 in Egypt, Turkey, Azerbaijan, Bangladesh, Iraq, Nigeria and Djibouti, and 2.3 in mainland China, Vietnam, Bangladesh, Laos, Canada and Myanmar, and 7 in mainland China. The data of clade was unavailable for four cases in Pakistan in 2007.

**Appendix Table 4. The characteristics of human case with H5N1 virus infection in Egypt before and since 1 November 2014**

| Characteristics | Total (n=363) | Before Nov 2014 (n=178) | Nov 2014 - Apr 2015 (n=185) | p value |
| --- | --- | --- | --- | --- |
| Type of cases | | | |  |
| Confirmed case | 342 (94.2%) | 177 (99.4%) | 165 (89.2%) | - |
| Probable case | 21 (5.8%) | 1 (0.6%) | 20 (10.8%) |  |
| Sex |  |  |  |  |
| Male | 144 (39.7%) | 72 (40.4%) | 72 (38.9%) | 0.965 |
| Female | 213 (58.7%) | 106 (59.6%) | 107 (57.8%) |  |
| Unknown | 6 (1.7%) | 0 (0) | 6 (3.2%) |  |
| Age | | | |  |
| Median (yrs, range) | 20 (0.25, 86) | 16 (0.25, 86) | 26 (0.6, 77) | 0.001 |
| Age group |  |  |  |  |
| 0-4 | 114 (31.4%) | 65 (36.5%) | 49 (26.5%) | Ref |
| 5-14 | 40 (11%) | 21 (11.8%) | 19 (10.3%) | 0.621 |
| 15-24 | 41 (11.3%) | 22 (12.4%) | 19 (10.3%) | 0.71 |
| 25-34 | 79 (21.8%) | 45 (25.3%) | 34 (18.4%) | 0.994 |
| 35-44 | 57 (15.7%) | 15 (8.4%) | 42 (22.7%) | <0.001 |
| 45-54 | 18 (5%) | 5 (2.8%) | 13 (7%) | 0.021 |
| 55-64 | 7 (1.9%) | 3 (1.7%) | 4 (2.2%) | 0.464 |
| 65 and above | 5 (1.4%) | 2 (1.1%) | 3 (1.6%) | 0.453 |
| Unknown | 2 (0.6%) | 0 (0) | 2 (1.1%) | - |
| Final outcome |  |  |  |  |
| Fatal | 116 (32%) | 64 (36%) | 52 (28.1%) | 0.125 |
| Non-fatal | 245 (67.5%) | 114 (64%) | 131 (70.8%) |  |
| Unknown | 2 (0.6%) | 0 (0) | 2 (1.1%) |  |
| Hospitalization | | | |  |
| Yes | 353 (97.2%) | 175 (98.3%) | 178 (96.2%) | - |
| No | 1 (0.3%) | 1 (0.6%) | 0 (0) |  |
| Unknown | 9 (2.5%) | 2 (1.1%) | 7 (3.8%) |  |
| Median of time delay (days, range) | | | |  |
| From onset to hospital admission | 3 (0, 33) | 2 (0, 13) | 4 (0, 33) | <0.001 |
| <3 days | 123 (33.9%) | 78 (43.8%) | 45 (24.3%) |  |
| ≥3 days | 194 (53.4%) | 74 (41.6%) | 120 (64.9%) |  |
| Unknown | 46 (12.7%) | 26 (14.6%) | 20 (10.8%) |  |
| From admission to outcome | 5 (0, 28) | 4 (0, 26) | 6 (0, 28) | 0.356 |
| Unknown | 219 (60.3%) | 110 (61.8%) | 109 (58.9%) |  |
| From onset to outcome | 10 (2, 32) | 8 (2, 30) | 11 (3, 32) | 0.044 |
| Unknown | 221 (60.9%) | 112 (62.9%) | 109 (58.9%) |  |
| Exposure history | | | |  |
| Any exposure to poultry | | | |  |
| No | 0 (0) | 0 (0) | 0 (0) | - |
| Yes | 339 (93.4%) | 171 (96.1%) | 168 (90.8%) |  |
| Unknown | 24 (6.6%) | 7 (3.9%) | 17 (9.2%) |  |
| Occupational exposure to live poultry | | | |  |
| No | 75 (20.7%) | 32 (18%) | 43 (23.2%) | 0.836 |
| Yes | 2 (0.6%) | 1 (0.6%) | 1 (0.5%) |  |
| Unknown | 286 (78.8%) | 145 (81.5%) | 141 (76.2%) |  |
| Visit LBMs | | | |  |
| No | 66 (18.2%) | 31 (17.4%) | 35 (18.9%) | 0.074 |
| Yes | 11 (3%) | 2 (1.1%) | 9 (4.9%) |  |
| Unknown | 286 (78.8%) | 145 (81.5%) | 141 (76.2%) |  |
| Exposure to sick or dead poultry | | | |  |
| No | 23 (6.3%) | 2 (1.1%) | 21 (11.4%) | <0.001 |
| Yes | 174 (47.9%) | 151 (84.8%) | 23 (12.4%) |  |
| Unknown | 166 (45.7%) | 25 (14%) | 141 (76.2%) |  |
| Exposure to backyard poultry | | | |  |
| No | 13 (3.6%) | 3 (1.7%) | 10 (5.4%) | 0.114 |
| Yes | 64 (17.6%) | 30 (16.9%) | 34 (18.4%) |  |
| Unknown | 286 (78.8%) | 145 (81.5%) | 141 (76.2%) |  |
| Human case contact | | | |  |
| No | 339 (93.4%) | 171 (96.1%) | 168 (90.8%) | 0.08 |
| Yes | 3 (0.8%) | 0 (0) | 3 (1.6%) |  |
| Unknown | 21 (5.8%) | 7 (3.9%) | 14 (7.6%) |  |

**
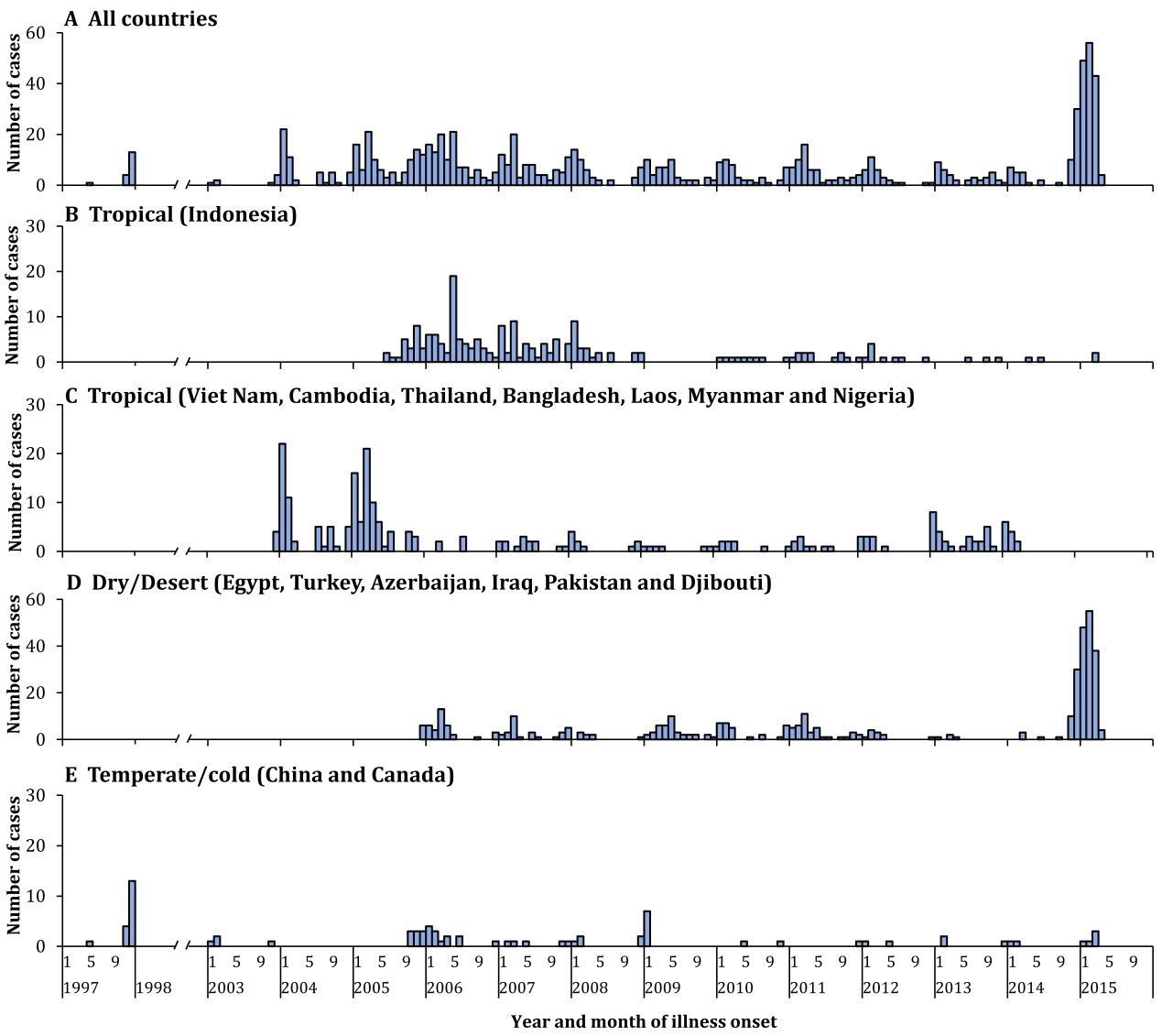
**

**Appendix Figure 1. Epidemic curve of human cases with H5N1 virus infection by climate zones, May 1997 – April 2015**

(A) The epidemic curve of H5N1 human cases reported globally (884 cases). (B) The epidemic curve of Indonesia (187 cases) in tropical area. (C) The epidemic curve of other tropical area (231cases) includes Vietnam (134), Cambodia (58), Thailand (27), Bangladesh (7), Nigeria (2), Laos (2), and Myanmar (1). (D) The epidemic curve of Dry/Desert area (390 cases) includes Egypt (363), Turkey (10), Azerbaijan (9), Pakistan (4), Iraq (3) and Djibouti (1). (E) The epidemic curve of temperate/cold area (76 cases) includes mainland China (52), Hong Kong SAR (23) and Canada (1). The case of Canada had a travel history in China before onset in 2013. Twenty-three cases with unknown month of illness (21 cases of Indonesia in 2009 and two cases of Turkey in 2006) are excluded from this figure. The climate zones were taken from the Koppen Climate Classification map of the world, and the map was available at <http://sedac.ciesin.columbia.edu/downloads/maps/nagdc/nagdc-population-landscape-climate-estimates-v2/Global_ClimateZone.jpg>.


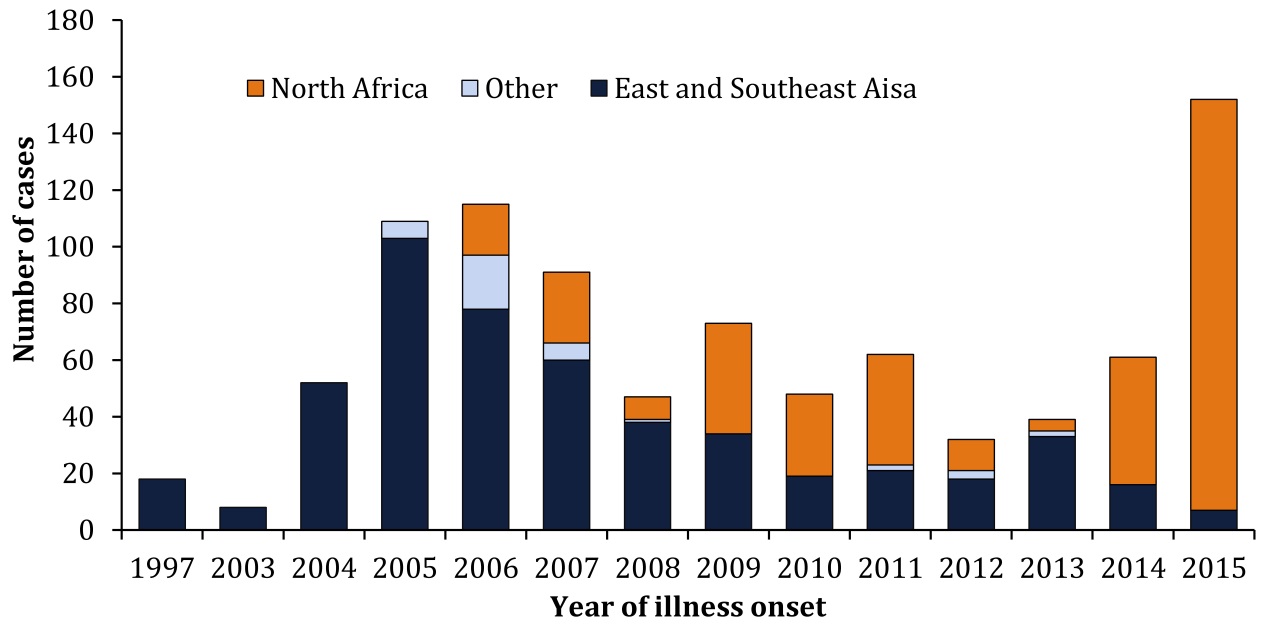


**Appendix Figure 2. The number of human cases with H5N1 virus infection by year and geographic region, May 1997 – April 2015 (n=907)**


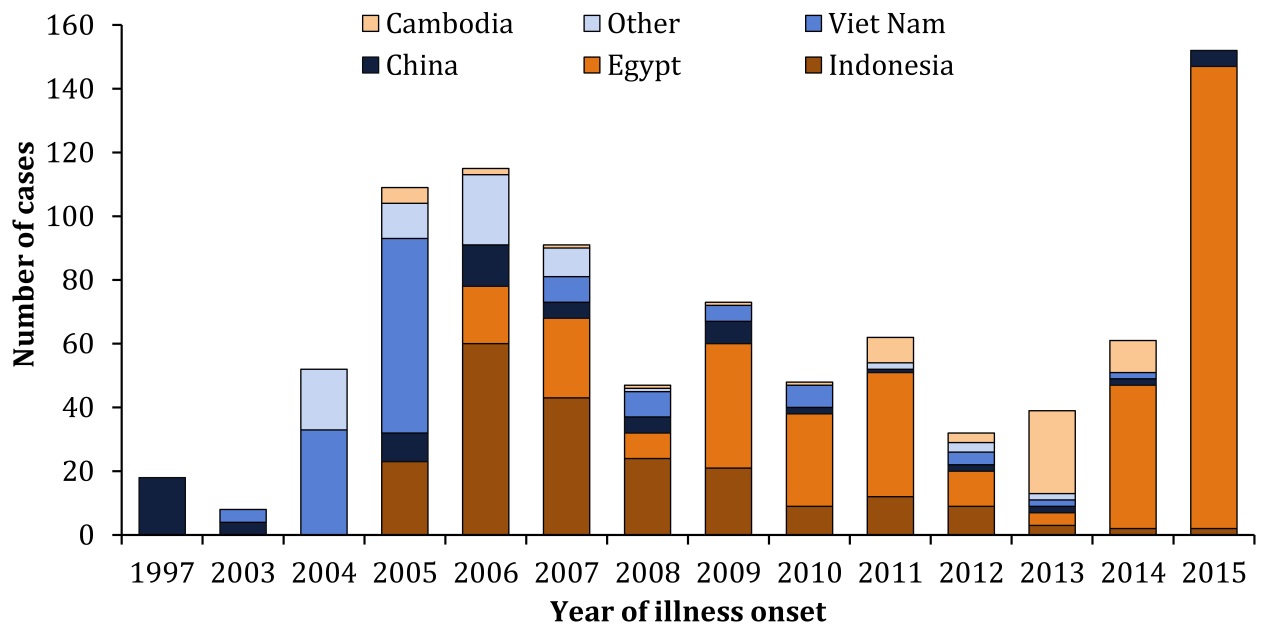


**Appendix Figure 3. The number of human cases with H5N1 virus infection by year and country,** **May 1997 – April 2015 (N=907)**


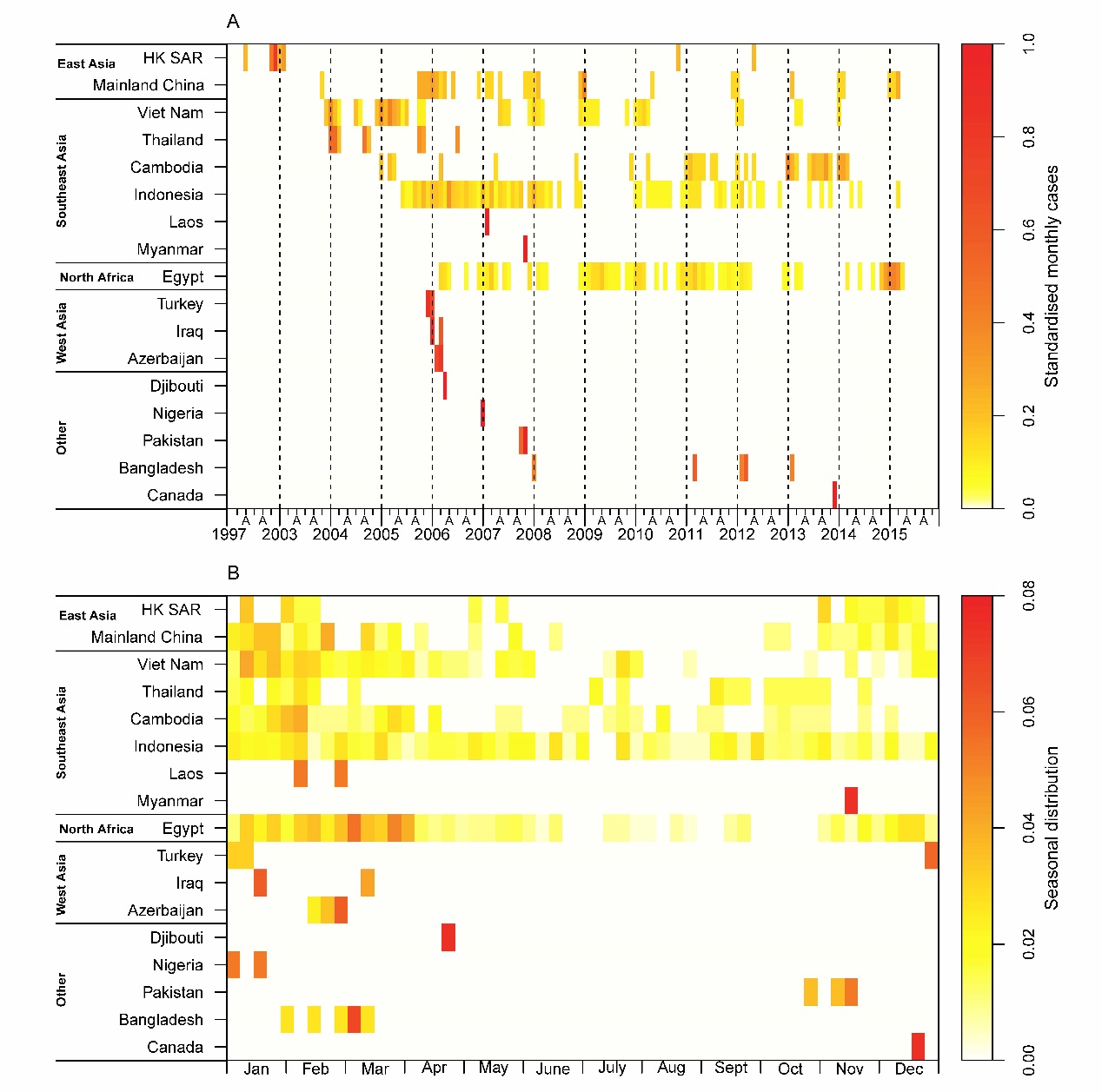


**Appendix Figure 4.** **Heat map of the reported data of human cases with H5N1 virus infection by country, sorted by geographical region and the date of the first cases illness onset, May 1997–April 2015.**

(A) Time series of monthly cases, standardized by the total number of cases in each country/region. Twenty-three cases with unknown month of illness (21 cases of Indonesia in 2009 and two cases of Turkey in 2006) are excluded. (B) Seasonal distribution of cases, plotted as the mean value of the proportion of weekly case number in the total number of each country/region. Forty-three cases with unknown week of onset are excluded: Indonesia (21 cases), Egypt (18), Turkey (2), Cambodia (1) and Vietnam (1). HK SAR: Hong Kong Special Administrative Region of China.


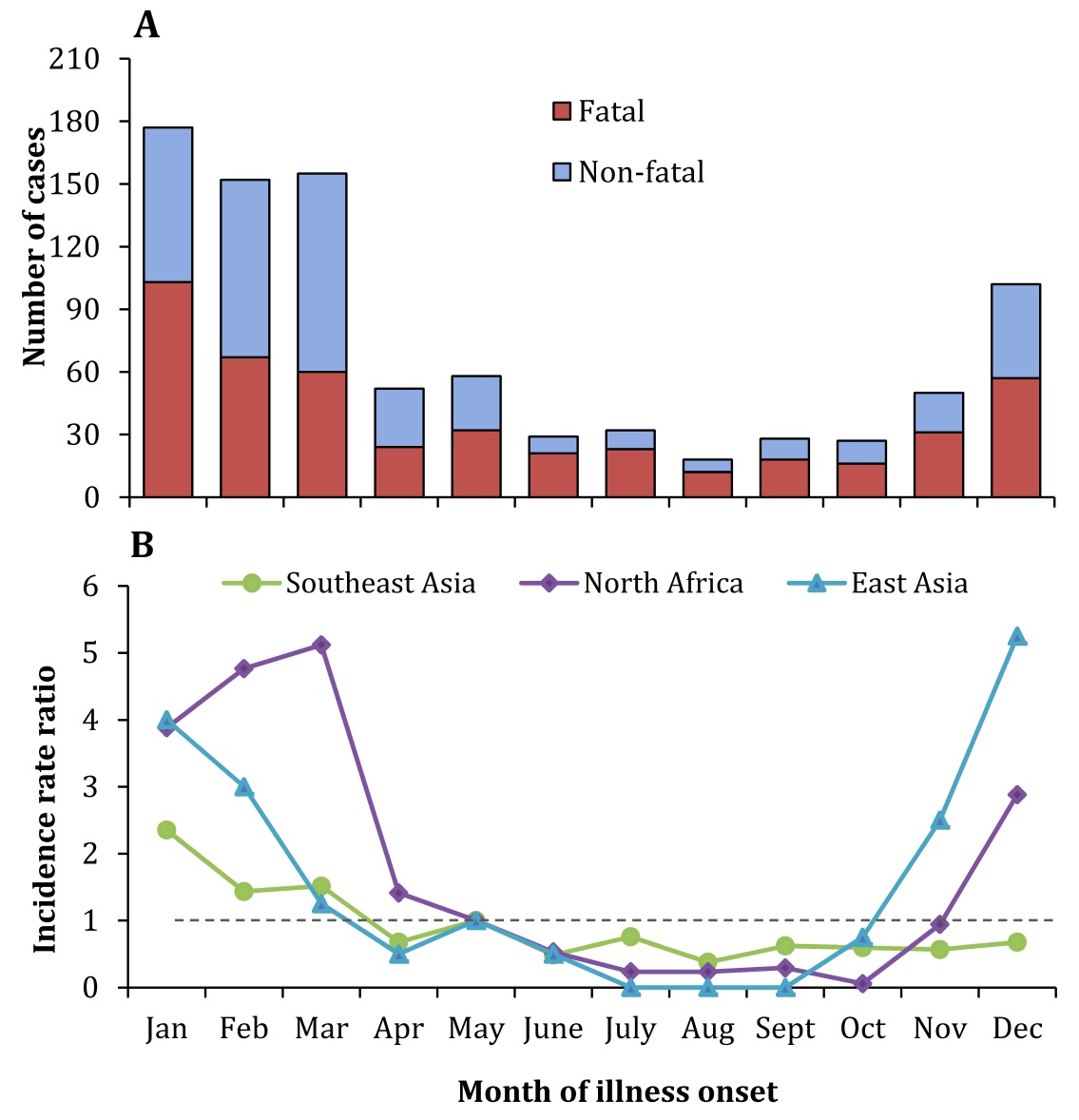


**Appendix Figure 5. The seasonality of human cases with H5N1 virus infection by the month of illness onset, May 1997 – April 2015**

(A) The number of monthly cases by outcome of all countries. (B): The incidence rate ratio of H5N1 infection in each month compared to May by region.


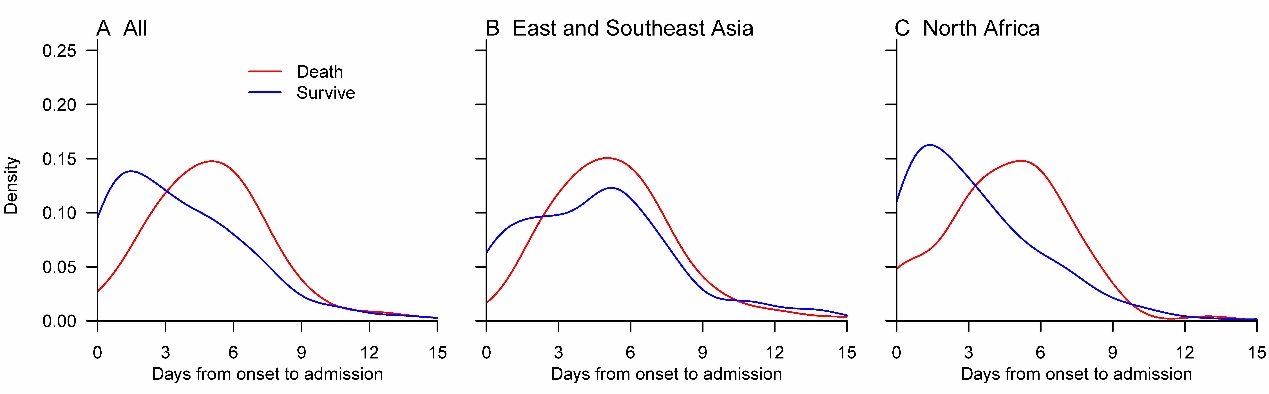


**Appendix Figure 6. The distribution of days from onset to hospital admission of human H5N1 cases by outcome and geographic region, May 1997–April 2015.**

The distribution of time intervals from onset of symptoms to hospital admission was presented and smoothed using kernel density estimation. (A) The time distribution of all fatal (n=380) and non-fatal (n=343) cases. (B) The time distribution of fatal (n=268) and non-fatal (n=116) cases in East and Southeast Asia. (C) The time distribution of fatal (n=99) and non-fatal (n=218) cases in North Africa.


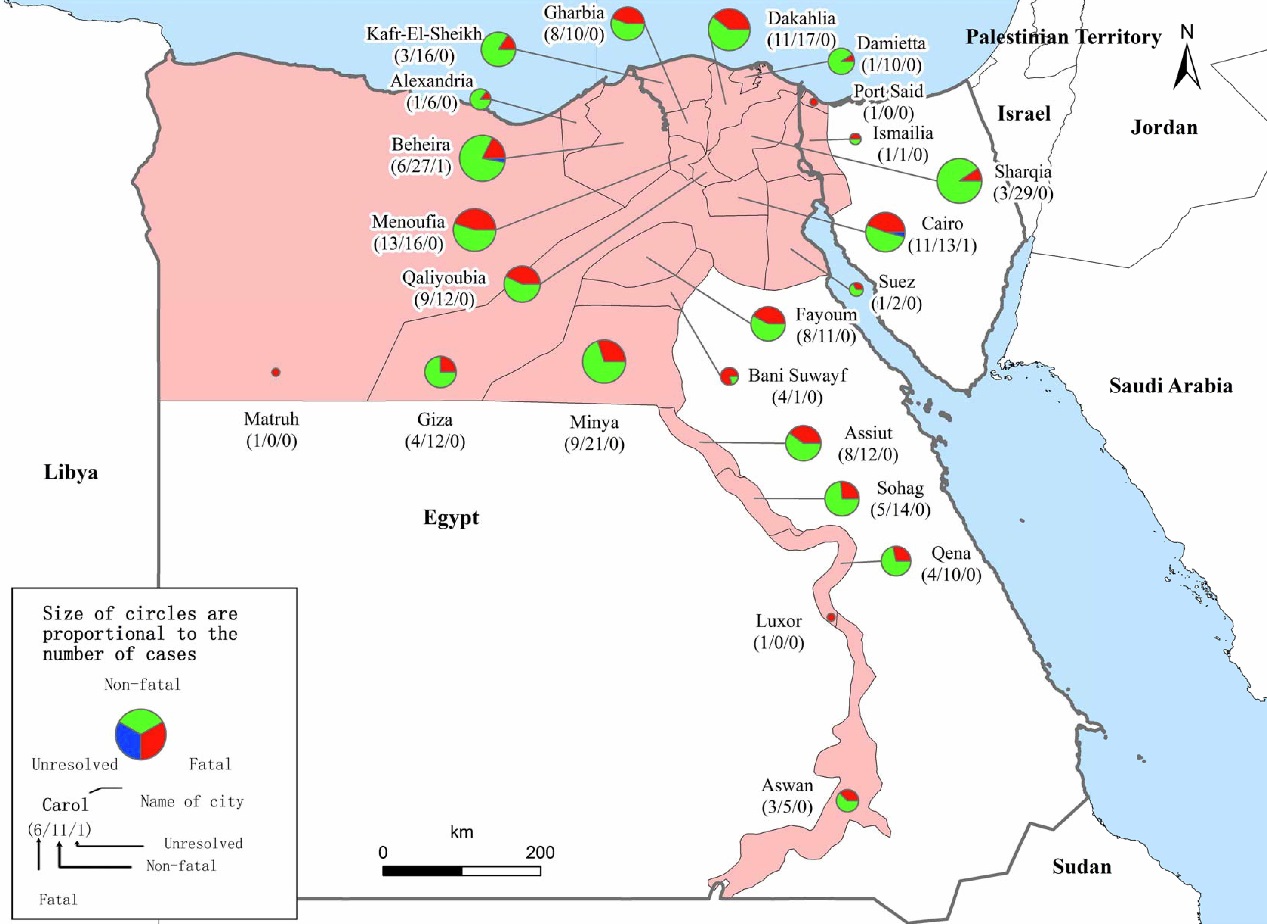


**Appendix Figure 7. The geographic distribution of human cases with H5N1 virus infection by outcome in Egypt, March 2006–April 2015 (n=363).**
